# Antifungals: Need to Search for a New Molecular Target

**DOI:** 10.4103/0250-474X.44588

**Published:** 2008

**Authors:** A. T. Sangamwar, U. D. Deshpande, S. S. Pekamwar

**Affiliations:** Department of Pharmaceutics, Nanded Pharmacy College, Nanded-431 605, India; 1 School of Life Sciences, S. R. T. M. University, Nanded-430 606, India; 2 Department of Pharmaceutical Chemistry, Nanded Pharmacy College, Nanded-431 605, India

**Keywords:** Antifungal agents, epidemiology, fungal infections, new targets, resistance

## Abstract

In the 1990s, drug resistance has become an important problem in a variety of infectious diseases including human immunodeficiency virus infection, tuberculosis, and other bacterial infections which have profound effects on human health. At the same time, there have been dramatic increase in the incidence of fungal infections, which are probably the result of alterations in immune status associated with the acquired immuno deficiency syndrome epidemic, cancer chemotherapy, and organ and bone marrow transplantation. The rise in the incidence of fungal infections has exacerbated the need for the next generation of antifungal agents, since many of the currently available drugs have undesirable side effects, are ineffective against new or reemerging fungi, or lead to the rapid development of the resistance. This review will focus on the pathogenic yeast *Candida albicans*, since a large body of work on the factors and mechanism associated with antifungal drug resistance in this organism is reported sufficiently. It will certainly elaborate the probable molecular targets for drug design, discovered to date.

Although extremely rare 10 years ago, antifungal drug resistance is quickly becoming a major problem in certain populations, especially those infected with HIV, in whom drug resistance of the agent causing oropharyngeal candidiasis is a major problem. For instance, 33% of late stage AIDS patients in one study had drug- resistant strains of *Candida albicans* in their oral cavity. There are no large scale surveys of the extent of antifungal drug resistance, which has prompted requests for an international epidemiological survey of this problem.

Treatment of invasive mycoses is complicated by the problems in diagnosis and susceptibility testing of fungi. This has consistently lagged behind bacterial chemotherapy. Amphotericin B, still the “gold standard” for the treatment of most severe invasive fungal infections, was discovered in 1956. One reason for the slow progress is that, like mammalian cells, fungi are eukaryotes, and thus, agents that inhibit protein, RNA, or DNA biosynthesis have greater potential for toxicity. A second reason is that until recently, the incidence of life threatening fungal infections was perceived as being too low to warrant aggressive research by the pharmaceutical industry. In the past decade, however, there has been a major expansion in the number of antifungal drugs available. Nevertheless, there are still major weaknesses in their spectra, potencies, safety, and pharmacokinetic properties. So the antifungal agents currently in use and the use of promising new biochemical targets in fungi are discussed here. This review will sufficiently open a path for researchers in this area to design and develop a new antifungal agent.

## FUNGAL INFECTIONS

Fungal infections have emerged during the past two decades as important pathogens causing formidable morbidity and mortality in an increasingly diverse and progressively expanding population of immunocompromised patients. Those with acquired immune deficiency syndrome (AIDS) constitute the most rapidly growing group of patients at risk for life threatening mycoses especially Cryptococcal meningitis^1^,^2^ disseminated histoplasmosis^3^ and coccidioidomycosis^4^,^5^. Oropharyngeal and oesophageal candidiasis are common infections in approximately one half of all children and adults with AIDS^6^,^7^.

Invasive candidiasis is one of the most common nosocomial mycoses (Centre for disease control, 1984). It is the most common fungal infection in patients with HIV infection, perhaps reflecting *Candida’s* niche as a component of the endogenous flora of the human alimentary tract. Conditions due to *Candida* species include, but are not limited to, oropharyngeal candidiasis^8^–^10^ esophageal candidiasis^11^, gastrointestinal candidiasis^12^,^13^, hematogenous *Candida* endophathalmitis^14^,^15^, hepatosplenic candidiasis^16^, renal candidiasis^17^, *Candida* cystitis^18^, epiglottitis^19^, Peritonitis^20^, cholecystitis^21^, prosthetic valve endocarditis^22^, vascular catheter- associated fungemia and thrombophlebitis^23^,^24^. Many infections due to C*andida* species are refractory to antifungal therapy. The patterns of resistance to amphotericin B may be classified as microbiological resistance or clinical resistance^25^.

## CURRENT ANTIFUNGAL AGENTS IN CLINICAL USE AND EXISTING TARGETS FOR DRUG DESIGN

Five major classes of systemic antifungal compounds are currently in clinical use; the polyene antibiotic, the azole derivatives, the allylamines, thiocarbamates and the fluoropyrimidines.

### Polyenes:

 The polyene antibiotics produced by *Streptomyces* species are fungicidal and have the broadest spectrum of activity of any clinical useful antifungal compound^26^,^27^. These compounds complex with ergosterol in the plasma membrane, causing membrane disruption, increased permeability, leakage of cytoplasmic contents and cell death^28^. Recent evidence suggests that they also cause oxidative damage, which may contribute to their fungicidal activity. The clinically useful polyenes, amphotericin B, nystatin, and natamycin (pimaricin) have a higher affinity for ergosterol than its mammalian counterpart cholesterol and are thus less toxic to mammalian cells^29^.

### Azoles:

 The azole derivatives discovered in the late 1960s, are totally synthetic and are the most rapidly expanding group of antifungal compounds^30^,^31^. They act primarily on ergosterol biosynthesis at the C-14 demethylation stage, a three step oxidative reaction catalyzed by the cytochrome P-450 enzyme, 14 alpha-sterol demethylase (P450_DM_). Azoles disrupt the structure of the plasma membrane, making it more vulnerable to further damage, and alter the activity of several membrane bound enzymes, such as those associated with nutrient transport and chitin synthesis^32^,^33^.

### Allylamines and Thiocarbamates:

 There are two allylamine antifungal agents, naftifine and terbinafine and one thiocarbamate, tolnaftate^34^. All three are reversible, noncompetitive inhibitors of squalene epoxidase^35^,^36^.

### Morpholines:

 The morpholines, discovered in the 1970s are totally synthetic and with the exception of amorolfine, which is used in the topical treatment of nail infections, are agricultural fungicides^37^. They also act on the ergosterol pathway, inhibiting two reactions, Δ14-reductase and Δ4-, Δ8-isomerase^38^.

### Flucytocine:

The fluoropyrimidine flucytosine (5-FC) has a limited spectrum of activity and is mainly used in combination with amphotericin B in Cryptococcal meningitis, as well as in case of disseminated candidiasis^39^.

## ANTIFUNGAL DRUG RESISTANCE–A FOCUS ON *CANDIDA*

Development of resistance to azole antifungal in *C. albicans* has been reported in the early 1980s in patients with congenital defects in their immune systems who were predisposed to chronic mucocutaneous candidiasis. This disease is an uncommon condition, but prolonged ketoconazole treatment leading to resistance in the infecting strains and clinical failure is well documented^40^–^42^. The increasing importance of the azole antifungal in the treatment of fungal infections has been matched by a growing interest in their mode of action and, more recently, in the mechanism by which C*andida* species can mutate to resistance. The antifungal drug resistance is discussed on three levels: 1) clinical factors that result in the inability to successfully treat refractory disease, 2) cellular factors associated with a resistant fungal strain; and 3) molecular factors that are ultimately responsible for the resistance phenotype in the cell. Many of the clinical factors that contribute to resistance are associated with the immune status of the patient, with the pharmacology of the drugs, or with the degree of type of fungal infection present. At a cellular level, antifungal drug resistance can be the result of replacement of a susceptible strain with a more resistant strain or species or the alteration of an endogenous strain to a resistant phenotype. The molecular mechanism of resistance that have been identified to date in *Candida albicans* include overexpression of two types of efflux pumps, overexpression or mutation of the target enzyme, and alteration of other enzymes in the same biosynthetic pathway as the target enzyme. Although extremely rare 10 years ago, antifungal drug resistance is quickly becoming a major problem in certain populations, especially those infected with HIV^43^,^44^. In late stage AIDS patients 33% had drug resistant strains of *Candida albicans*^45^, needs international epidemiological survey^46^.

### Epidemiology:

It appears that ampotericin B resistance in *Candida* spp. and *Cryptococcus neoformans* can develop in patients previously exposed to azole antifungal agents due to an alteration of cellular membrane components 47–53. Primary resistance to 5-FC is common in certain yeasts and molds. Non-C. *albicans* spp., as well as *Aspegillus* spp., *C. neoformans*, and the dimorphic fungi, have high rates of 5-FC resistance^54^,^55^. In addition, secondary resistance is a common development especially in patients receiving 5-FC monotherapy. The prevalence of azole resistance has been estimated to be 21 to 32% in symptomatic patients and up to 14% in asymptomatic patients^56^–^58^. Azole resistance is due to defects in drug import, modification or degradation of the drug, modification of ergosterol biosynthetic pathway or molecular alteration of the ERG11 gene. Allylamine resistance has not been reported for medically important fungi, although resistant strains have been described in *S. cerevisiae* and the plant pathogen^59^.

## NEED FOR SEARCHING NEW TARGETS

Treatment of deeply invasive infections has consistently lagged behind bacterial chemotherapy^60^. It is complicated by problems in diagnosis and susceptibility testing of fungi^61^–^63^. Fungal infections are increasingly important health threat as the number of immunocompromised individuals continues to rise. The recent surge in the use of antifungal agents, particularly azoles, is selecting resistant strains of susceptible species and is shifting the population of fungal pathogens towards species that are intrinsically resistant. The conditions that have led to the emergence of fungal infections in the past 10 years are likely to persist in the future. New approaches are urgently needed for improved diagnosis, including species identification, rapid and predictive susceptibility assays, and effective treatment

A limited repertoire of effective antifungal drugs with acceptably low host toxicity has motivated the search for novel antifungal drug target. But the identification of a target unique to fungi has been a challenge, given the remarkable similarity between fungal and mammalian metabolic and signal transduction pathways. There are however, several promising antifungal target evaluations under active investigation. Enzymes involved in the biosynthesis of the fungal cell wall, lipid composition of the plasma membrane, and DNA and protein synthesis have been targeted, with various degrees of success^64^.

### Strategies in designing a new target:

Existing antifungal agents either kill a range of fungal pathogens or considerably retard their growth, yet they are not always clinically successful. Many authors argue a case for virulence factors as antifungal targets, antifungal agents are usually required after such factors have done their work and tissues are already infected. Virulence factors are therefore conceptually more likely to be targets for prophylactic rather than therapeutic agents, and may be highly specific to a single species or strains within a species. Comparative analyses of fungal genomes and molecular research on genes associated with fungal viability and virulence has led to the identification of many putative targets for novel antifungal agents. So far the rational approach to antifungal discovery, in which compounds are optimized against an individual target then progressed to efficacy against intact fungi and ultimately to infected humans has delivered no new agents. However, the genomic approach continues to hold promise for the future.

## NEW TARGETS FOR ANTIFUNGAL AGENTS

### Fungal Cell Wall:

Rational drug design is limited to well-characterized targets and mechanistically understood reactions, in which structure optimization, including computer-aided modeling is feasible. The fungal cell wall, a structure essential to fungi and lacking in mammalian cells, is an obvious target for antifungal agents^65^,^66^. Its major macromolecular components are chitin, B-glucan, and mannoproteins^67^–^69^.

### Plasma Membrane:

The fungal plasma membrane contains sterols and phospholipids as its major lipid components and functions as a permeability barrier, conduit for the transport of small molecules and signals and a matrix for proteins. Anchored to or embedded into the membrane are proteins whose co-or post translational modification may also yield therapeutic targets.

### Ergosterol Synthesis:

Most rational drug design efforts have focused on fungal sterols since they are structurally distinct from their mammalian counterparts and their biosynthesis has been studied extensively^70^–^72^.

### Phospholipid Synthesis:

Fungal phospholipids are synthesized by pathways that are basically similar to their mammalian counterparts^73^,^74^.

### Sphingolipid Synthesis:

Sphingolipids are essential membrane components of both mammalian cells and fungi and are localized primarily on the other leaflet of the fungal cytoplasm membrane^75^.

### Proton ATPases:

The plasma membrane H^+^ ATPases is an integral membrane protein belonging to the P-type class of ion-translocating ATPases. It is an abundant essential enzyme involved in the maintenance of electrochemical proton gradients and the regulation of intracellular pH. Plasma membrane ATPases are known in sufficient molecular detail to be targets for rational drug design^76^.

### Efflux Pumps:

Proteins with pump function have been reported in *Candida* species^77^–^79^, and may be responsible for the observed broad resistance of these organisms to azoles and perhaps to other antifungal agents. Recent studies suggest fungal topoisomerase I can be inhibited selectively^80^.

### Protein Synthesis:

Both fungal and mammalian cells require two soluble protein factors, elongation factor EF-1 alpha and EF-2 for the polypeptide chain elongation reactions of protein synthesis^81^. However fungi require an additional factor, EF-3, which is absent from mammalian cells^82^,^83^.

### Intermediary Metabolism Nucleic Acid:

The success of trimethoprim–sulphomethoxazole in treating *P. carinii* pneumonia has validated their sites of action in the folate pathway as drug targets for this organism although not for other fungi^84^.

### Aminoacids:

The discovery of the amino acid analog cispentacin^85^, an antifungal agent with excellent *in vivo* activity^86^ and multiple cellular targets^87^ raised the possibility of interfering with amino acid synthesis.

### Polyamines:

Ornithine decarboxylase, the rate limiting enzyme in polyamine synthesis^88^, and a favorite target in anticancer chemotherapy^89^, may also be an antifungal target.

### Other Cellular Functions Microtubules:

Microtubules are dynamic polymers of alpha and beta-tubulin dimers^90^. They form a highly organized cellular skeleton in all eukaryotic cells, and their aggregation–disaggregation plays a key role in cell morphology and growth. Microtubule aggregation is inhibited by griseofulvin, the agricultural fungicide benomyl, and the anticancer drugs vincristine and vinblastine; desegregation is inhibited by taxol^91^.

### Signal Transduction and Cell Cycle:

Yeasts, in particular *S. cerevisiae* and *S. pombe*, have been extensively used as models for intracellular signal transduction and the cell cycle of mammalian cells^92^–^96^.

### Virulence Factors:

Virulence factors in medically important fungi are best defined by molecular genetic approaches in which putative virulence genes are examined by deletion and transformation.

### Genes:

*Candida* genes have ability to induce hyphae formation under selected conditions^97^. Virulence properties of *C. albicans* includes adherence to host cells and the ability to undergo the transition from yeast to hyphal growth known as dimorphism^98^,^99^.

### Histidine Protein Kinase, N-myristoyltransferase and Aspartic Proteinase:

Histidine protein kinases are potential antifungal drug targets. The pathogenic fungus *C. albicans* harbors three histidine kinase genes called CaSLN1, CaN1Kl, and CaHK1. The disruption of any one of these three genes impairs the hyphal formation and attenuates the virulence of *C. albicans*. Potent and selective *C. albicans* N-myristoyltransferase (CaNmt) inhibitors are identified through optimization of a lead compound. Secreted aspartic proteinases (SAPs) are important virulence factors in different types of candidiasis caused by *C. albicans.*

## SELECTION OF NEW TARGETS

Development of new approaches for treatment of invasive fungal infections encompasses new approved and investigational compounds exploiting the ergosterol biosynthesis pathway, cell membrane, cell wall and virulence factors as putative antifungal targets.

### Ergosterol Biosynthesis Inhibition:

The major product of sterol biosynthesis in fungi is ergosterol, unlike in mammalian systems which synthesize cholesterol as the major membrane lipid^100^,^101^. Prelanosterol Steps are acetate to mevalonate, mevalonate to squalene 2,3-oxidosqualene cyclase. Post lanosterol steps include those that play a role once lanosterol has been formed by the cyclization of squalene epoxide, it undergoes several sequential transformations to form ergosterol. The exact route that the sterol molecule follows depends on the availability of the substrate and the specific interactions between the individual enzyme and that substrate in a given fungus. The different steps are Cl4 demethylation, C14 reductase, C7-C8 isomerase, 24-methenylation, and C4 demethylation.

### Late Stages:

There are no known inhibitors of the later stages of the pathway that are antifungal. This may be because the organisms are more tolerant to the small changes in the structure of the ring, as results with trifluperidol in *C. albicans*^102^. The development of resistance in fungi to fungicides with specific mechanisms of action is quite common. Clinical isolates of pathogenic organisms less susceptible to the azoles have however, appeared during treatment with ketoconazole^103^. The use of inhibitors of ergosterol biosynthesis in the treatment of fungal infections is still in its infancy. At present, the C14 demethylase inhibitors, the azoles, are the major therapeutic agents in clinical use.

### Virulence Associated Mannoproteins of *Candida albicans:*

The cell surface of *C. albicans* is the subject of considerable study over the past fifteen years^104^. There is a static arrangement of three polysaccharides (glucan, chitin and mannan), lipid and some protein. The mannoproteins can be growth-form specific i.e. found exclusively on germ tubes^105^,^106^, variable in expression, found in high numbers on exponential but not in stationary phase yeast cells^107^,^108^.

### Adhesins of *Candida albicans:*

Adherence to host tissues is important as a first step in systemic infection. Since colonization by *Candida* species is a necessary prerequisite for the establishment of certain infections. The specific attachment of microorganisms is mediated by surface constituents called adhesins. The virulence determinants of *Candida* include the ability to form hyphae^109^, to resist phagocytosis^110^, and to produce extra cellular hydrolytic enzymes such as proteinases^111^, and phospholipases^112^.

### Complement receptors:

Sheep red blood cells (E) which are sensitized with anti-SRBC antibody (A) are used as a matrix to generate intermediates in the complement cascade. The primary important complement C3 ligands are EAC3b, EAiC3b, and EAC3D. It is established that *C. albicans* possesses cell surface binding proteins which recognize the ic3b and c3d ligands but not the c3b ligand^113^,^114^. There is a role of ic3b receptor in allowing *C. albicans* to escape phagocytosis^115^.

### Laminin and Fibrinogen Binding Proteins:

*C. albicans* possesses cell-surface proteins which bind fibrinogen^116^–^118^ as well as laminin. There is a correlation between adherence of the organism to the buccal mucosal surface and differential expression of secretory proteinase by different *Candida* species.

### Enzyme Topoisomerase II inhibitors:

The DNA of both prokaryotic and eukaryotic cells exhibits several common tertiary structures including supercoils, knots and catenanes, each of which is involved in various cellular processes^119^–^121^. These topological forms of DNA may arise as a result of it being in a closed, circular form, such as in plasmid, viral, phage or bacterial genomic DNA. The topoisomerases carry vital cellular functions in DNA replication, DNA repair, chromosome segregation, ribosomal RNA synthesis, transcription and sister chromatid exchange^122^,^123^. Camptothecin is the classical topoisomerase inhibitor and stabilizes DNA enzyme cleavage complex^124^.

## DEVELOPMENT OF HIGH THROUGHPUT SCREENS

An ideal screen allows a high throughput of samples. It obeys general law that, technically simple, economic to operate, requires only small amounts of compounds, easy and unambiguous to read and sensitive enough not to miss potential positives.
